# Comparison of regeneration capacity and *Agrobacterium*-mediated cell transformation efficiency of different cultivars and rootstocks of *Vitis* spp. via organogenesis

**DOI:** 10.1038/s41598-018-37335-7

**Published:** 2019-01-24

**Authors:** S. Sabbadini, L. Capriotti, B. Molesini, T. Pandolfini, O. Navacchi, C. Limera, A. Ricci, B. Mezzetti

**Affiliations:** 1 Department of Agricultural, Food and Environmental Sciences, Università Politecnica delle Marche, Ancona, Italy; 2 Department of Biotechnology, University of Verona, Verona, Italy; 3 Vitroplant International, Cesena, Italy

## Abstract

The success of *in vitro* plant regeneration and the competence of genetic transformation greatly depends on the genotype of the species of interest. In previous work, we developed a method for the efficient *Agrobacterium*-mediated genetic transformation via organogenesis of *V*. *vinifera* cultivar Thompson Seedless, by using meristematic bulk (MB) as starting tissue. In this study, we applied this method for the regeneration and transformation of MBs obtained from the Italian cultivar Ciliegiolo and two of the commonly used *Vitis* rootstocks, 110 Richter and Kober 5BB, in comparison with Thompson Seedless. The *A*. *tumefaciens* strain EHA105, harbouring pK7WG2 binary vector, was used for the transformation trials, which allowed selection through the enhanced-green fluorescent protein (eGFP) and the neomycin phosphotransferase (*nptII*) gene. Putative transformed tissues and/or shoots were identified by either a screening based on the eGFP expression alone or its use in combination with kanamycin in the medium. MBs obtained from Thompson Seedless showed the highest regeneration and transformation cell competence, which subsequently allowed the recovery of stably transformed plants. Ciliegiolo, 110 Richter, and Kober 5BB, produced actively growing transgenic calli showing eGFP fluorescence, more consistently on selective media, but had no regenerative competence.

## Introduction

Genetic engineering of grapevine represents a powerful alternative to conventional plant breeding programs, offering the possibility of introducing useful agronomic traits in cultivars and/or rootstocks without altering their desirable characteristics. Until now, the breeding of grapevine has been mainly centred on the introgression from donor parents of genes capable of improving biotic stress resistance^1^. However, the practical application of conventional breeding methods for grapevines has proved to be problematic mainly because of the long-juvenile period and reproductive cycle, high level of heterozygosity and the introgression of undesirable genes during crossbreeding that can affect the agronomic performance and quality of a cultivar^2,3^. Genetic transformation could help to overcome these drawbacks. A prerequisite for successful genetic transformation of any plant species is the availability of an efficient and reproducible genotype-specific *in vitro* regeneration protocol, which has become one of the major bottlenecks in grapevine research^1,4^. Grapevine *in vitro* regeneration has been mainly obtained by the induction of adventitious shoot regeneration through somatic embryogenesis from different explants, such as anther filaments^1^. The use of anther filaments as a starting material for somatic embryogenic calli induction requires high expertise for the selection of the correct flower stage and is characterized by several drawbacks, such as time consuming and laborious processes for the creation and maintenance of the embryogenic cultures. In addition, this method is not applicable to some grapevine genotypes^5^. In contrast, protocols have been developed for grapevine *in vitro* regeneration by direct or indirect organogenesis from adult tissues, including leaves and petioles^6–10^, apical fragments of shoots^11,12^ and internodes^13,14^. We also obtained high regeneration efficiency via organogenesis starting from meristematic bulk (MB) tissue, obtained by culturing mechanically-treated shoot tips on media containing increasing concentrations of cytokinins^15^. The MB tissue showed a high meristematic/regenerative competence capable of producing a high number of shoots. Consequently, this method could be suitable for either vegetative plant propagation or for genetic transformation. Slices prepared from the MB tissue were used for *Agrobacterium*-mediated transformation of grapevine plants using the ovule-specific auxin-synthesizing (*DefH9-iaaM*) transgene^15–17^ that enhances fecundity in grapes, thus enabling an increase of yield with lower production costs^18^. The same protocol was later successfully applied for the *in vitro* regeneration and the genetic transformation of different *V*. *vinifera* cultivars, such as Chardonnay, Redglobe, Cabernet Sauvignon, and *V*. *rupestris* rootstock St. George^19,20^, as well as for other fruit species such as *Prunus* spp. and blueberry^21–23^.

Regeneration and transformation efficiencies are a function of the plant species genotype^2,24^, and are influenced by different factors including the kind of starting explant, the *Agrobacterium* strain used for infection, and the selective agents added to the culture medium^1^. An effective selective regime is of primary importance in selecting the transformed tissues and for eventually regenerating transgenic plants. Sensitivity to the presence of antibiotic-selective agents in the medium strongly affects the regeneration efficiency and consequently the production of transformed plants^25^. In this regard, necrosis and tissue browning caused by reactive oxygen production, induced after *Agrobacterium* inoculation, which is commonly observed in some genotypes, could be a response to the effects of the selective agent, imposing downstream effects on the regeneration capacity^25–28^. The employment of non-destructive marker genes, such as the green fluorescent protein (GFP), represents an efficient selective strategy. The use of non-destructive reporter genes alone or combined with antibiotic-based selection system might ease the identification of escapes and chimera, which are commonly protected when the selection system relies solely on the presence of antibiotics in the culture medium. This phenomenon has often been associated with the persistence of antibiotic-resistant *A. tumefaciens* cells, and/or to the presence of transformed plant cells close to non-transformed ones with a supposed detoxifying effect in the surrounding area^24,29–32^. Visual screening with GFP can be exploited as a unique method for selection of transgenic events, which helps in the reduction of negative effects on organogenesis related to the addition of antibiotics in the culture medium. This could also help to avoid the use of selectable marker genes such as antibiotic-resistance genes, which raise concerns related to their possible negative effects on the environment and human health^24,29,31–37^. The use of reporter genes has been widely investigated both for the optimization of grapevine transformation protocols, and for functional genomic studies of this species^20,38,39^. Recently, a *V*. *vinifera*-derived sequence corresponding to the *MybA1* gene has been used as a cisgenic reporter for the selection of grapevine transformed lines derived from somatic embryogenic cultures^40,41^. This study underlines the importance of identifying new plant-derived genes as reporters and/or selectable markers for isolation of transformed plants^41^.

Another factor affecting transformation efficiency is the *A*. *tumefaciens* strain used. There are several strains available for transformation trials but, among these, EHA105 strain is the most commonly employed in grapevine transformations^5,42–46^.

In this study, we applied the protocol based on the meristematic bulk induction^15^ for the genetic transformation of a *V*. *vinifera* local cultivar, Ciliegiolo, and two rootstocks 110 Richter (*V*. *berlandieri* x *V*. *rupestris*) and Kober 5BB (*V*. *berlandieri* x *V*. *riparia*). The responses of the three genotypes to *in vitro* regeneration and transformation protocols were evaluated in comparison to the regeneration/transformation efficiency of Thompson Seedless. The *A*. *tumefaciens* strain EHA105, harbouring pK7WG2 binary vector, was used for the transformation trials. The T-DNA of the pK7WG2 binary vector contained enhanced-green fluorescent protein (*eGFP*) reporter gene and neomycin phosphotransferase (*nptII*) gene that confers resistance to kanamycin. We compared the combined use of the *nptII* gene and eGFP visual screening to the use of eGFP as a unique means for the recovery of transformed events and plants obtained via organogenesis.

## Results

### Regeneration capacity of MBs and sensitivity to kanamycin for the optimization of the selection protocol

**Figure 1:**
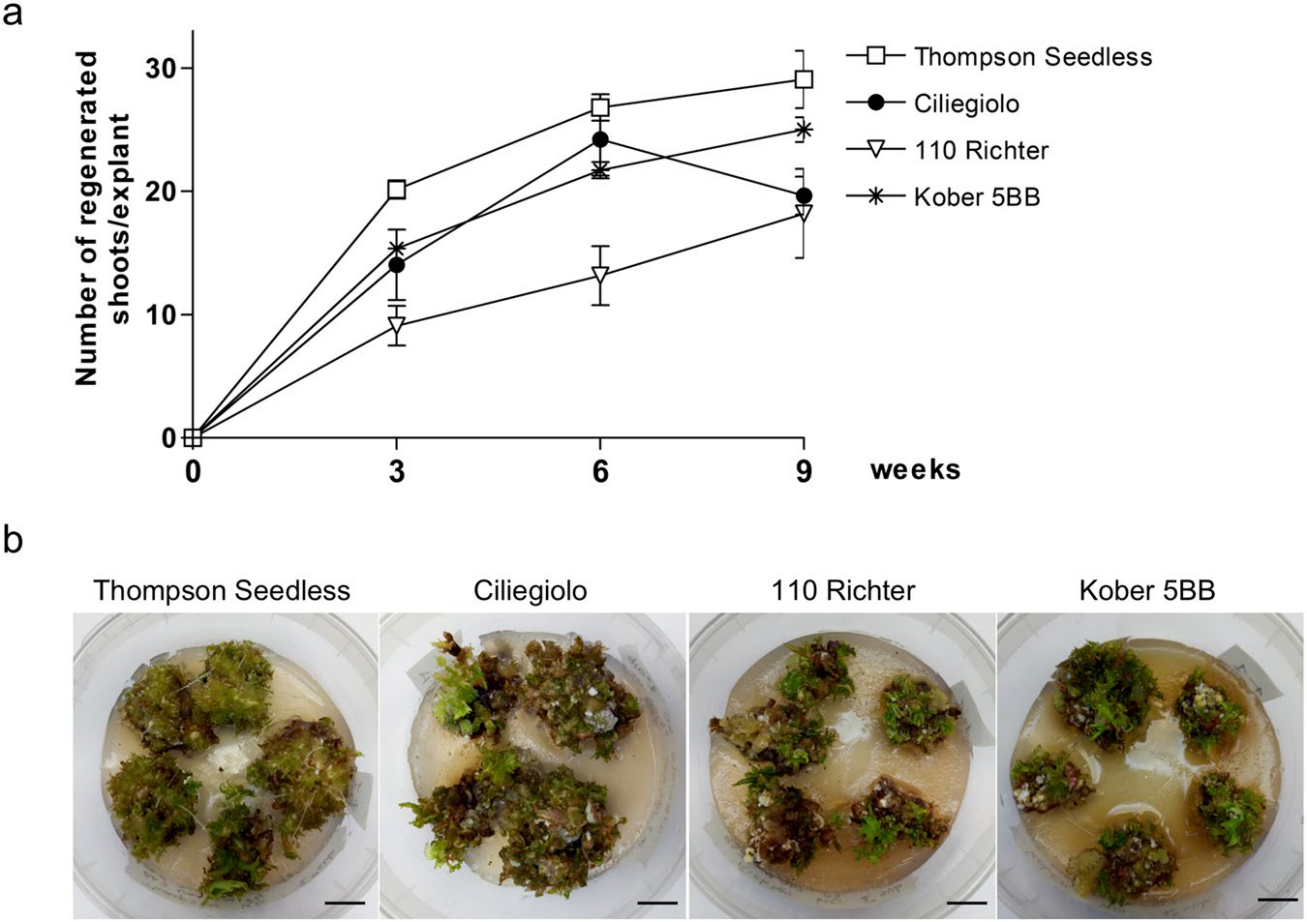
Regeneration efficiency obtained from non-transformed MB slices of Thompson Seedless, Ciliegiolo, 110 Richter and Kober 5BB: (**a**) Mean number of shoots per explant. Data were acquired at 3, 6 and 9 weeks of cultures. Data reported are means of ± SE (n = 20). (**b**) A representative image of the MB slices regeneration of the four genotypes on IM3 medium, kanamycin-free, after three weeks of culture (*bar* = 1 cm).

Before performing the transformation trials, a regeneration test was conducted for the variety Ciliegiolo, and for the two rootstocks, 110 Richter and Kober 5BB. Thompson Seedless variety, that has been efficiently transformed by us^15^, was used as the comparative genotype. MB slices obtained from the four genotypes were placed on IM3 medium without kanamycin, and data on the regeneration efficiency (percentage of slices producing at least one shoot, data not shown) and the mean number of regenerating shoots per explant were collected at 3, 6 and 9 weeks of cultivation (Fig. 1a). All the genotypes showed a comparable regeneration efficiency with values close to 100% of regenerating slices (data not shown), starting from 3 weeks of culture and maintained for the entire culture period (Fig. 1b). The rootstock Kober 5BB showed the highest mean number of regenerated shoots per explant with values comparable to those exhibited by Thompson Seedless (Fig. 1a). 110 Richter’s MB slices exhibited the lowest regeneration efficiency in terms of mean number of shoots per explant throughout the data acquisition periods (Fig. 1a). Ciliegiolo showed the same trend as Thompson Seedless and Kober 5BB until 6 weeks of cultivation, after which the number of regenerated shoots declined (Fig. 1a).

To establish the concentration of kanamycin for the selection of putative transformed lines, MB slices derived from the four grapevine genotypes were placed on IM3 culture medium containing increasing concentrations of kanamycin (i.e. 50 mg L^−1^, 70 mg L^−1^, and 100 mg L^−1^) (Table 1). As expected, an increase in the concentration of kanamycin in the medium led to a gradual decline in the regeneration efficiency as well as the number of regenerated shoots per explant. In addition, a progressive necrosis of the meristematic tissues and the regeneration of bleached shoots was observed (Supplementary Fig. 1). MB slices of 110 Richter showed the lowest sensitivity to kanamycin in the culture medium whereas Kober 5BB showed the highest.

**Table 1:** Kanamycin sensitivity of non-transformed MB slices of Thompson Seedless, Ciliegiolo, 110 Richter and Kober 5BB.

	Kan mg L^−1^	Thompson Seedless		Ciliegiolo		110 Richter		Kober 5BB	
		N° green shoots/explant	MBR(%)	N° green shoots/explant	MBR(%)	N° green shoots/explant	MBR(%)	N° green shoots/explant	MBR(%)
3 weeks	50	4.7 ± 1^(A,a)^	95 ± 5^(A,a)^	3.2 ± 1.26^(A,a)^	60 ± 18.26^(A,a)^	6.4 ± 0.80^(A,a)^	100 ± 0^(A,a)^	3.45 ± 1^(A,a)^	85 ± 15^(A,a)^
	70	3.4 ± 1^(A,a)^	75 ± 9.57^(B,a)^	1.20 ± 0.48^(A,b)^	65 ± 12.58^(A,a)^	2.3 ± 0.24^(B,ab)^	75 ± 5^(B,a)^	0.65 ± 0.15^(B,b)^	50 ± 5.77^(B,a)^
	100	—	—	0.05 ± 0.05^(A,a)^	5 ± 5^(B,a)^	0.1 ± 0.06^(B,a)^	10 ± 5.77^(C,a)^	—	—
6 weeks	50	1.45 ± 0.88^(A,b)^	25 ± 12.58^(A,b)^	0.65 ± 0.22^(A,b)^	40 ± 8.16^(A,b)^	3.80 ± 0.42^(A,a)^	80 ± 8.16^(A,a)^	0.80 ± 0.57^(A,b)^	15 ± 9.57^(A,b)^
	70	0.60 ± 0.54^(A,a)^	15 ± 9.57^(A,ab)^	0.05 ± 0.05^(A,a)^	5 ± 5^(B,ab)^	0.90 ± 0.44^(A,a)^	25 ± 5^(B,a)^	—	—
	100	—	—	—	—	—	—	—	—
9 weeks	50	0.90 ± 0.71^(A,a)^	20 ± 14.14^(A,a)^	0.05 ± 0.05^(A,a)^	5 ± 5^(A,a)^	0.55 ± 0.32^(A,a)^	30 ± 17.32^(A,a)^	—	—
	70	0.1 ± 0.1^(A,a)^	5 ± 5^(A,a)^	—	—	0.1 ± 0.1^(A,a)^	5 ± 5^(A,a)^	—	—
	100	—	—	—	—	—	—	—	—

Meristematic Bulk regeneration efficiency (MBR%), expressed as a percentage of total number of MB slices treated ± SE (n = 20); n°of green shoots regenerating expressed as mean number per explant ± SE (n = 20). Data were analysed using one-way ANOVA and the Newman-Keuls test. Significant (p < 0.05) differences are indicated with different letters. Values followed by capital letters (A, B, C) compare the same genotype cultured on different kanamycin concentrations at the same acquisition time; while small letters (a,b,c) compare different genotypes at the same kanamycin concentration and acquisition time. Two independent experiments were performed with similar results. Data reported in the table are related to a unique trial.

All the genotypes showed an early (3 weeks of culture on kanamycin at 50 mg L^−1^) significant reduction in number of green shoots regenerated per explant (Table 1), compared to their regeneration on medium without kanamycin (Fig. 1a,b). Significant differences in terms of percentage meristematic bulk regeneration (MBR%) became visible only after placing the tissues on 70 mg L^−1^ kanamycin after 3 weeks of culture (Table 1, and Supplementary Fig. 1). Ciliegiolo explants were the only exception exhibiting significant sensitivity to kanamycin at this concentration, starting from 6 weeks of culture. Regeneration efficiency was almost completely arrested in all the genotypes on kanamycin 70 mg L^−1^ after 9 weeks of culture (Table 1). A marked toxicity effect became visible after 3 weeks of culture of MB slices on kanamycin at 100 mg L^−1^ (Supplementary Fig. 1), with a dramatic drop of both the regeneration efficiency and number of green regenerated shoots per explant. Thus, kanamycin at the concentration of 70 mg L^−1^ was chosen as the most appropriate for the selection method based on kanamycin resistance, to be used after *Agrobacterium*-mediated transformation of the four genotypes. This is because, it allows the regeneration of MB slices during the first weeks of culture, but completely inhibits the growth of non-transformed calli and shoots at a later time of *in vitro* culture.

### Transformation competence of the *Agrobacterium*-infected MBs screened via eGFP combined to kanamycin selection or with eGFP alone

**Figure 2:**
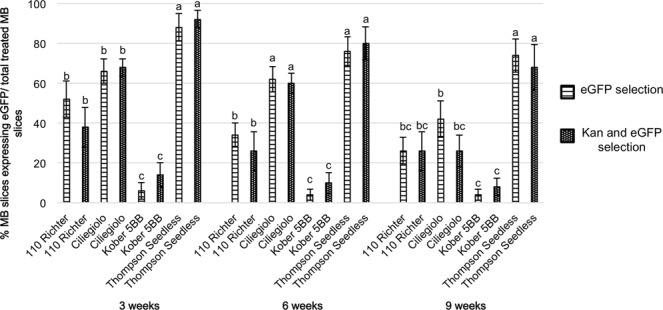
Percentage of MB slices showing eGFP fluorescence, selected either by the combined use of kanamycin and eGFP screening or by eGFP visual selection alone, after 3, 6 and 9 weeks on culture media. Means with different letters are significantly different according to Newman-Keuls test (p < 0.05) ± SE (n = 50), and compare results obtained from the different genotypes at the same acquisition period. Two independent experiments were performed with similar results. Data reported in the table are related to a unique trial.

After *Agrobacterium* infection, half of the explants of each genotype was transferred onto IM3 culture medium supplemented with kanamycin (70 mg L^−1^), while the remaining half was placed on the same medium but without the selective agent. The MB slices were transferred onto fresh media every three weeks and visual screening of transformed cells using eGFP was conducted at each subculture to detect and dissect the putative transformed areas and/or proliferating shoots. The percentage of MB slices showing eGFP fluorescence is reported in Fig. 2. Generally, except for MB slices from 110 Richter, calli sections fluorescing after three weeks on both culture conditions (i.e medium supplemented with or without kanamycin), continued to stably express eGFP during the whole culture period. Compared to the other genotypes, Thompson Seedless exhibited the highest percentage of transformed calli areas at almost all data acquisition periods, with final recorded values of 68% and 74% of eGFP-fluorescent explants when cultured on media supplemented with or without kanamycin respectively. Kober 5BB showed the lowest percentage of eGFP-fluorescent calli sectors at three weeks of culture, corresponding to 6% and 15% on media with and without kanamycin respectively, and maintained this trend during the whole culture period.

**Figure 3:**
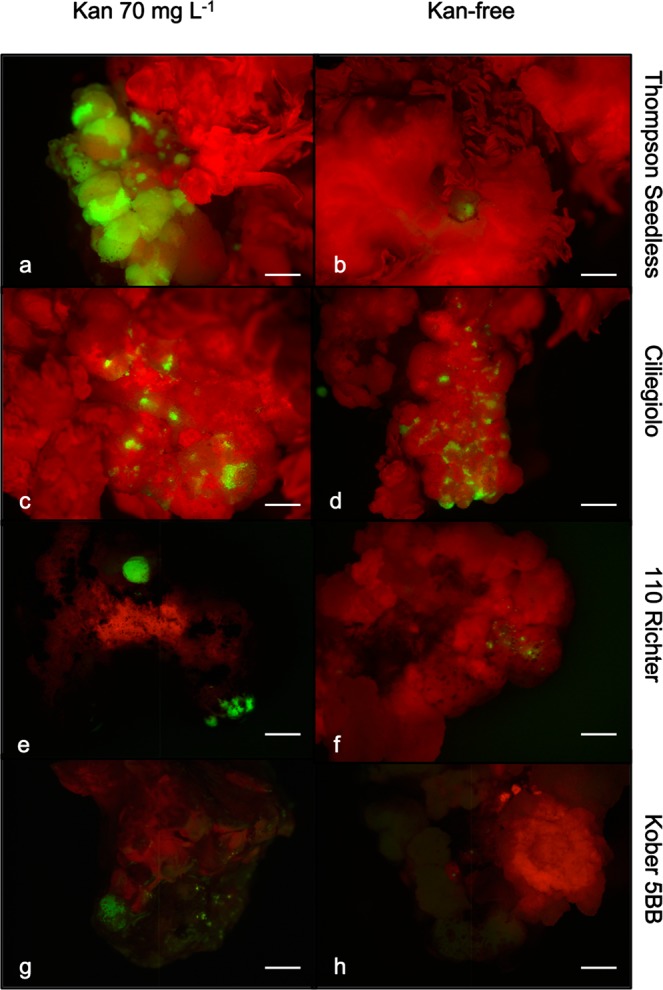
eGFP fluorescence detection in *Agrobacterium*-infected MB slices of Thompson Seedless (**a**,**b**), Ciliegiolo (**c**,**d**), 110 Richter (**e**,**f**) and Kober 5BB (**g**,**h**) after 3 weeks on medium supplemented with 70 mg L^−1^ kanamycin (left column), or kanamycin-free (right column) (*bar* = 2 mm).

**Figure 4:**
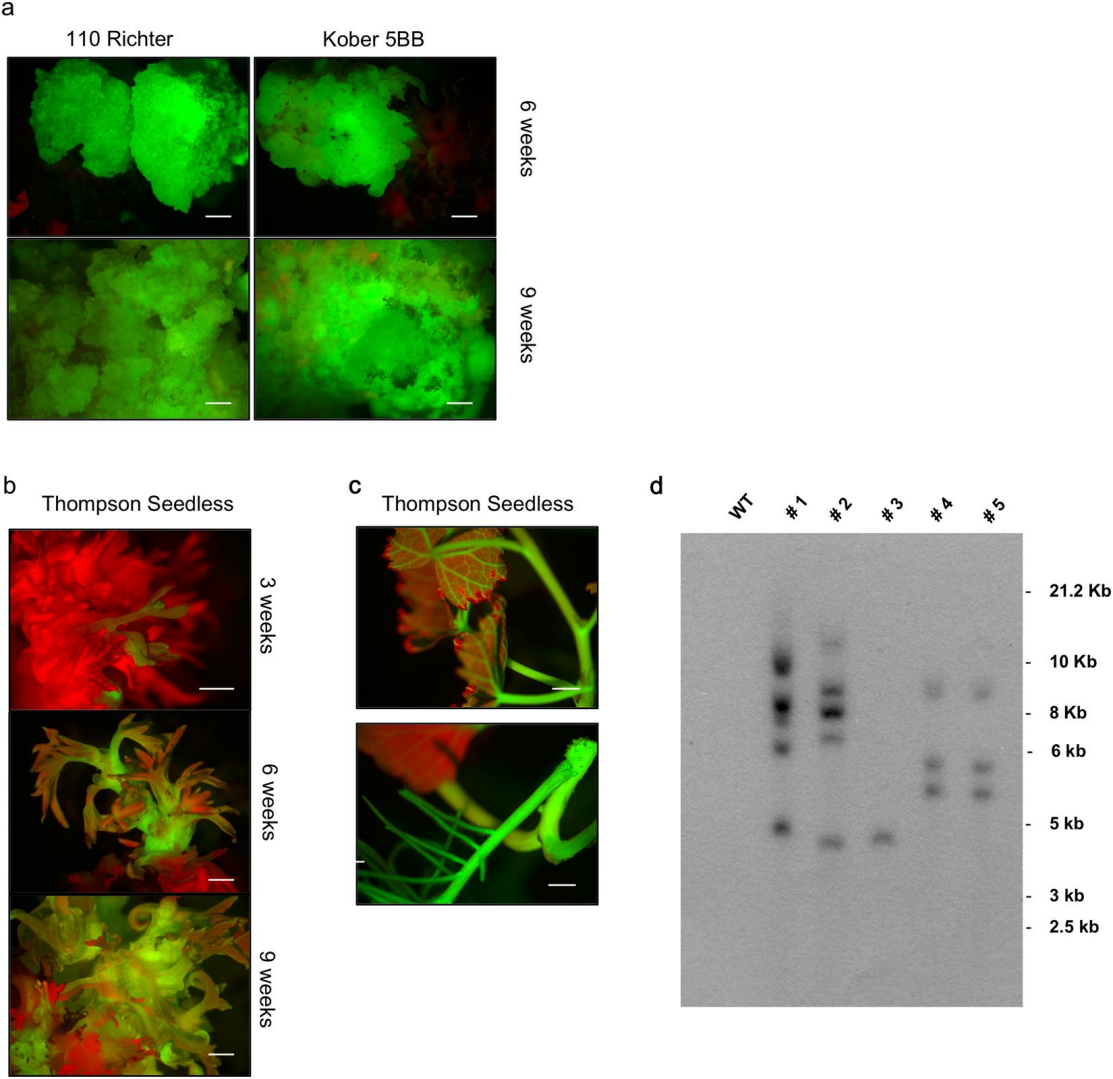
eGFP-detection and transgenic state evaluation in different grapevine genotypes: (**a**) 110 Richter (left column) and Kober 5BB (right column), after 6 weeks and 9 weeks on kanamycin 70 mg L^−1^; (**b**) Thompson Seedless transgenic shoot regeneration at 3, 6, 9 weeks on kanamycin 70 mg L^−1^; (**c**) Thompson Seedless elongated and rooted transgenic shoot. Uniform fluorescence with bright green colour was observed in transformed tissues under UV light (*bar* = 2 mm); (**d**) Transgenic state of T0 35 S::eGFP grapevine transgenic plants evaluated by Southern blot. 20 μg of genomic DNA from control non-transformed plant (lane 1) and transgenic lines #1, #2, #3, #4, and #5 (lanes 2–6, respectively) was digested with *Hind*III. The image was obtained after overnight exposure. Full-length blot was presented in Supplementary Fig. 3.

Although the frequency of MB slices expressing eGFP did not show a substantial variation when explants were cultured in the presence or absence of kanamycin in the medium (Fig. 2), the extension of the eGFP-expressing sections on the total MB surface displayed significant differences, especially for Thompson Seedless and 110 Richter early in the experiment (at 3 weeks of culture) (Fig. 3, and Table 2). Thompson Seedless MBs displayed uniform and extended fluorescing areas, which represented 28.6% of the total MB surface, when cultured on media containing kanamycin 70 mg L^−1^, a value more than five times higher than that obtained when explants were cultured in the absence of kanamycin at the same time period (Fig. 3a,b, and Table 2). Ciliegiolo exhibited small green fluorescing spots scattered on the whole MB surface, surrounded by non-transformed cells on both media supplemented with kanamycin and without (Fig. 3c,d). This led to difficult mechanical dissection of the eGFP-expressing areas from the non-transformed surrounding cells for the entire period of selection (9 weeks). The two rootstocks, 110 Richter and Kober 5BB displayed small areas emitting eGFP fluorescence at 3 weeks of culture, which were uniform on MB slices cultured on kanamycin selection (Fig. 3e,g), allowing an easier visual and mechanical isolation of the transgenic areas from the non-transformed ones. These tissues proliferated and expanded during the whole culture period on kanamycin 70 mg L^−1^, thus producing homogenous conspicuous green fluorescing calli, which were unable to regenerate shoots (Fig. 4a). However, MB calli sections from the two rootstocks exhibited the same trend observed in Ciliegiolo (i.e. limited extended area on the total MB slice surface, Fig. 3f,h) when cultured on kanamycin-free media. This displayed chimeric events throughout the culture period, which was not observed on the same tissues cultured with selective agent. The presence of kanamycin in IM3 medium also helped in reducing the regeneration of escapes; nonetheless, some explants from Thompson Seedless, in this culture condition, continued to regenerate non-transformed shoots often situated close to eGFP-expressing calli area (Supplementary Fig. 2a).

**Table 2:** Percentage of eGFP-derived green fluorescing area on MB slices cultured for 3 weeks on media supplemented with 70 mg L^−1^ kanamycin (Kan) or without (Kan-free).

Genotype	% of eGFP area/total MB area	
	Kan 70 mg L^−1^	Kan-free
Thompson Seedless	28.6^(a)^	5.7^(b)^
110 Richter	9.97^(b)^	<0.001^(c)^
Ciliegiolo	6.16^(b)^	5.48^(b)^
Kober 5BB	0.005^(c)^	<0.001^(c)^

Means (n = 3) with different letters are significantly different according to Newman-Keuls test (p < 0.05).

Thompson Seedless was the only genotype that regenerated transgenic shoots (Fig. 4b,c): ten lines from MB slices selected on 70 mg L^−1^ kanamycin were recovered, some of which were isolated after the first 3 weeks of selection (Fig. 4b), and one transgenic shoot regenerated after 9 weeks of culture on medium without kanamycin (Supplementary Fig. 2b). The putative transformed shoots were isolated and transferred on rooting medium. Four of the ten lines (i.e #1, #3, #4, and #5) obtained through the combined use of eGFP and kanamycin selection and the #2 line obtained by the eGFP visual selection only, were able to produce roots *in vitro*. Southern blot analysis revealed that line #3 contained one copy of the transgene; lines #1 and #2, had five and four copies respectively (Fig. 4d). The remaining two lines, # 4 and #5, displayed the same pattern of hybridization signals (i.e. 3 copies of the transgene), thus representing a single transformation event (Fig. 4d).

## Discussion

The regeneration and transformation protocol via organogenesis, developed as previously described^15^, was applied for the first time to the local wine grape cultivar Ciliegiolo, and to two rootstocks, 110 Richter and Kober 5BB, and was compared with the table grape cultivar Thompson Seedless.

To our knowledge there are no reports on the *in vitro* regeneration of explants from Ciliegiolo, and few studies exist on the *in vitro* regeneration by organogenesis of explants of the two rootstocks^14,47–49^, for which somatic embryogenesis is the preferred regeneration system^50–52^.

The *Agrobacterium*-mediated transformation protocol applied in this study led to the production of numerous transgenic calli and/or shoots for all the genotypes tested. However, differences in the transformation competence and efficiency of the different cells were observed. Introduction of the genetic cassette expressing *eGFP* reporter gene has revealed several advantages for the detection of putative transgenic events and the discrimination of escapes during the whole culture period in both selective and non-selective conditions. Furthermore, the use of eGFP screening combined with the addition of kanamycin in the culture medium proved to be a useful selection system. Even though the addition of kanamycin in the medium didn’t affect the percentage of transgenic events observed in the four genotypes (Fig. 2), it influenced the eGFP area extension on calli surfaces. This allowed the production of larger eGFP-fluorescing calli sections, compared to the same tissues placed on non-selective condition (Fig. 3). Indeed, it was not possible to obtain similar consistently proliferating transformed calli from MB tissues cultured on non-selective media, probably due to the absence of selection pressure normally conferred by kanamycin on the non-transformed cells. From the results obtained, it also seems that the transformation efficiency is not linked to the initial regeneration potential; MB slices of Ciliegolo and Kober 5BB didn’t produce any transgenic shoots after transformation, although they showed regeneration efficiency comparable to that of Thompson Seedless before *Agrobacterium* infection. Thompson Seedless confirmed its high regeneration and transformation efficiency as already observed by us and other studies^12,15,20,25,53–55^. MB slices belonging to this genotype were the only plant material that regenerated transgenic shoots, on both selective and non-selective conditions. Stably transformed cells were obtained from MB slices of 110 Richter and Kober 5BB in both culture conditions (i.e. presence or absence of kanamycin in the medium); however, the eGFP-expressing calli sections cultured on kanamycin showed a higher proliferating ability although without shoot regeneration competence, which led to the isolation of big stable eGFP-fluorescing calli (Fig. 4a). Similar findings were previously reported^29^ on transformation of apple cultivars; a decrease of approximately 10.000-fold of transgenic shoots regeneration was observed after *Agrobacterium* infection, despite the high starting regenerative capability of the genotypes treated. It has been reported that the production of transgenic plants relies on the simultaneous capability of the cell to integrate the *Agrobacterium* T-DNA and to regenerate shoots, which mainly depends on the genotype, the phytohormones present in the culture medium, and their mutual interaction during transformation^29,56–58^. Different studies show how the regeneration and/or transformation cell efficiency is strongly regulated by several factors, such as expression of plant specific genes/transcription factors, the phase of cell cycle at the time of transformation (the regeneration of transgenic shoots has been positively correlated to the frequency of S and G2 nuclei presence), and the capability of de-differentiated transgenic cells to revert back to the production of an embryo or a meristem (the so called “stem-cell-like state”) (reviewed by Arias *et al*. 2006). The majority of Thompson Seedless transgenic shoots recovered didn’t seem to initiate from globular like intermediate cluster of calli (Supplementary Fig. 2c), but from compact, translucent meristematic cells (Supplementary Fig. 2d), with both regeneration and transformation competences. The absence of transgenic shoot regeneration from the other genotypes could be attributed to the fact that the transgene seemed to only integrate in the cells lacking regenerative competence. We showed in our previous work that the internal part of the MB tissue is composed by hyper-trophic parenchymatous cells, with highly vascularized bands, which include initiation nodules from which adventitious shoots originate^15^. We can suppose that the transgene often integrates in these types of cells, which could also be present in the MB slices of Ciliegiolo, Kober 5BB and 110 Richter. However, further histological analyses on these genotypes are necessary to confirm this hypothesis.

In conclusion, the results obtained on regeneration efficiency demonstrate that the MB system is a relatively simple and fast method for increasing the efficiency of *in vitro* regeneration in a wide range of grapevine genotypes, in accordance with previous data^15,20^. The use of *eGFP* as reporter gene confirmed its value in understanding the processes of T-DNA introgression and regeneration efficiency of the different grapevine genotypes. Further studies need to be performed to adjust the culture conditions and phytohormonal medium composition for the regeneration of MB slices of Ciliegiolo, Kober 5BB, and 110 Richter to be used during the extensive culture period after *Agrobacterium* infection. Moreover, the application of specific pre- and post-*Agrobacterium* infection treatments (e.g. exposure of the starting explants to a preliminary dark condition phase, the use low *A*. *tumefaciens* densities for tissues infection, the optimization of the conditions of the source material to be used for transformation and the addition of virulence genes from *A*. *tumefaciens* to increase transformation frequency) could enhance the competence of cells for both regeneration and transformation in these genotypes^12,34,57,59,60^. The novel achievement of this study is the description of an easy method to produce, via organogenesis, stably and actively proliferating transgenic calli of two widely used grapevine rootstocks. These materials can represent a valuable tool for studying genetic and molecular features that control the transformation and regeneration capacity as well as for gene functional analysis. Thompson Seedless has been confirmed to be a model grapevine genotype that can be exploited for future functional gene studies. In our research we demonstrated that, although with a low frequency, there is the possibility for easy-to-transform grapevine genotypes to obtain transgenic plants via organogenesis with the use of eGFP visual screening only, hence avoiding the antibiotic selection. This protocol can also be optimized for the introduction of grapevine-derived reporter gene systems to obtain cisgenic plants, which could help in reducing some of the concerns associated with genetically modified crops.

## Material and Methods

### Plant material

*In vitro* proliferating shoots of *Vitis vinifera* cultivar Ciliegiolo, Thompson Seedless and the two rootstocks, 110 Richter (*V*. *berlandieri* x *V*. *rupestris*) and Kober 5BB (*V*. *berlandieri* x *V*. *riparia*), were provided by Vitroplant Italia S.r.l., Cesena, Italy, and used as starting plant material for the induction of meristematic bulks and genetic transformation trials. The vegetative material was subcultured every 30 days on a medium containing MS salt and vitamins^61^, 3% sucrose, 4.4 μM of 6-benzylaminopurine (BAP) and 7 g L^−1^ plant agar. The explants were kept in a growth chamber at 24 ± 1 °C under a photoperiod of 16-h light (70 μmol m^−2^ s^−1^) provided by white fluorescent tubes.

### MB induction and maintenance

The MB induction and maintenance was obtained following the protocol previously described^15^. Briefly, shoot tips derived from proliferating cultures were processed and placed on the initiation medium (IM) described previously^15^, with the addition of 0.01 μM 1-Naphthaleneacetic acid (NAA). At each monthly subculture (a total of 3), the apical dome was eliminated and the concentration of BAP in the IM medium was increased: 4.4 μM (IM1), 8.8 μM (IM2), and 13.2 μM (IM3). The obtained MBs were maintained and proliferated by slicing them on IM3 culture medium and used as starting materials for the subsequent regeneration and transformation experiments. The explants were kept in a growth chamber at 24 °C under a photoperiod of 16-h light (70 μmol m^−2^ s^−1^) provided by white fluorescent tubes.

### *Agrobacterium* strain and grapevine MBs transformation conditions

*A*. *tumefaciens* strain EHA105 harbouring pK7WG2 binary vector was used for the transformation experiments. The T-DNA of the pK7WG2 binary vector contained the *eGFP* and the *nptII* genetic cassette. The transformation protocol reported previously^15^ was used including slight modifications. Bacterial cultures were inoculated in liquid YEB medium (5 g L^−1^ of yeast extract, 1 g L^−1^ of peptone, 5 g L^−1^ sucrose, 490 mg L^−1^ MgSO_4_, pH 7.2) containing 50 mg L^−1^ rifampicin, and 75 mg L^−1^ spectinomycin and grown at OD_600_ = 0.5–1.0 at a temperature of 28 °C on a shaker at 200 rpm. The bacteria was pelleted and re-suspended in MS20 liquid medium (4.4 g L^−1^ of MS salts including vitamins, 2% sucrose, pH 5.2), supplemented with 100 μM acetosyringone. Grapevine MBs were cut into slices 1 cm^2^ and 2 mm thick and dipped in the bacterial suspension for 15 min, blotted on filter paper and subsequently transferred onto MSH0 solid medium (4.4 g L^−1^ of MS salts including vitamins, 3% sucrose, 0.7% plant agar, pH 5.7). The plates were incubated for 48 hours in dark condition at 24 °C.

### Regeneration efficiency and MBs sensitivity to kanamycin

Before starting with the transformation trials, the toxicity threshold of kanamycin was determined by placing non-transformed MB slices (1 cm^2^, 2 mm thick) of each grapevine genotype in microboxes (Micropoli, IT) containing IM3 medium supplemented with 300 mg L^−1^ cefotaxime and kanamycin 0, 50, 70, and 100 mg L^−1^. The explants were transferred on fresh media every 3 weeks for a total of three subcultures, cutting off the new regenerating shoots at each subculture in order to simulate the mechanical treatments normally applied after *Agrobacterium* infection. A total of 20 MB slices, divided in four replicates, were placed on each selection medium. The experiment was repeated twice. Regeneration response of MB slices on each medium was monitored recording the following data: percentage of regenerating MB slices [(number of MB slices regenerating shoots/total MB slices treated) × 100], and mean number of green shoots regenerated from each explant at each culture condition. All the data were recorded after each sub-culture (at 3, 6 and 9 weeks). Furthermore, the threshold of toxicity of the selective agent was evaluated through the observation of the effects caused by kanamycin on the phenotype of regenerating shoots compared to the respective control (MB slices placed on IM3 kanamycin-free, with the same number of replicates).

### Identification of putative transgenic lines using eGFP alone or in combination with kanamycin for selection

A total of 100 MB slices of each genotype were utilized for the transformation trial; in parallel *Agrobacterium* non-inoculated MB slices were placed on IM3 and used for regeneration efficiency comparative purposes. After the co-culture with *Agrobacterium*, 50% of the explants for each genotype were placed in microboxes containing fresh IM3 medium supplemented with 300 mg L^−1^ of cefotaxime to contain *Agrobacterium* survival, and 70 mg L^−1^ of kanamycin. The remaining 50% was placed on fresh IM3 medium not supplemented with kanamycin. The explants were transferred on fresh media every three weeks, and observed under the stereomicroscope Leica MZ10F to detect calli sectors and shoots emitting green fluorescence (λ_EX_ = 480 nm and λ_EM_ = 510 nm). Images were acquired by the Leica DFC 450 C camera and processed through the Leica Application Suite V.4.5. At each subculture (3, 6 and 9 weeks), new regenerating shoots and calli areas not fluorescent were cut and discarded, and only putative transgenic zones were transferred to fresh media. The number of MB slices expressing eGFP were counted and expressed as percentage of the total explants infected. The experiment was repeated twice.

### Imaging analysis to quantify transformed area

Green fluorescent areas showing eGFP fluorescence were analysed through the ImageJ software (https://imagej.nih.gov/ij/) and quantified through the colour threshold function, which measures a specific coloured area of the image and expresses it as pixel value. The results reported are the mean of three different measures of three different MB calli chosen randomly for each genotype at the two culture conditions (IM3 with or without kanamycin). The results obtained were expressed as percentage of the total surface of MB calli after three weeks of culture on media with or without the addition of kanamycin.

### Acclimatization of putative transgenic lines

Regenerating shoots showing stable eGFP green fluorescence were isolated and placed on IM medium supplemented with 4.92 μM Indole-3-butyric acid (IBA) where they stayed for a month. Then, *in vitro* rooted clones were transferred in the greenhouse and acclimatized in 350 cc pots containing commercial peat.

### Genomic DNA extraction and Southern blot analysis of Thompson Seedless transgenic plants

Genomic DNA was isolated from 0.5–1 g of frozen leaves using the “Illustra DNA extraction kit PHYTOPURE” (GE Healthcare) according to the manufacturer’s instructions. Then, genomic DNA was cleaned up using NucleoSpin Plant II (Macherey-Nagel). The DNA (20 μg) was digested with 40 U of *Hind*III in the presence of spermidine (5 mM) and BSA (0.1 mg/ml), then electrophoresed on a 0.8% agarose gel at 4.5 V cm^−1^, and transferred on positively charged Hybond N^+^ membrane (GE Healthcare). DNA probe for the analysis, spanning a 341 bp-long portion of the 35 S promoter used to drive eGFP expression, was obtained by PCR with the following forward (F) and reverse (R) primers: F 5′-CTTCGTCAACATGGTGGAGCACGACA-3′ and R 5′-TGGAGATATCACATCAATCCACTTG-3′. The probe was labelled with dCTP [α ^32^P] (Perkin Elmer) by random priming using “Prime-It II Random Primer Labeling Kit” (Stratagene). Unincorporated nucleotides were removed with the “Illustra AutoSeq G-50 Dye Terminator Removal Kit” (GE Healthcare). After overnight hybridisation at 42 °C in ULTRAhyb buffer (Ambion) in the presence of 10^6^ cpm ml^−1^ of labelled probe, the blot was washed twice in 2X SSC containing 0.1% SDS at 42 °C for 5 min, and twice in 0.1XSSC containing 0.1% SDS at 42 °C for 15 min. Autoradiography was then performed using CL-XPosure film (Thermo Scientific).

### Statistical analyses

All acquired data from each trial were analysed by one-way ANOVA, and Newman-Keuls test (p** < **0.05) was used to identify significant differences. The results obtained from both regeneration and transformation trials were analysed.

## Supplementary information

Supplementary Figures

## Data Availability

No datasets were generated or analysed during the current study.

## References

[1] Saporta R, Pedro-Galan S, Domenech G, Carmen M (2016). Attempts at grapevine (Vitis vinifera L.) breeding through genetic transformation: the main limiting factors. Vitis..

[2] Rai MK, Shekhawat NS (2014). Recent advances in genetic engineering for improvement of fruit crops. Plant Cell, Tissue and Organ Culture..

[3] Limera C, Sabbadini S, Sweet JB, Mezzetti B (2017). New biotechnological tools for the genetic improvement of major woody fruit species. Frontiers in plant science..

[4] Gray DJ, Li ZT, Dhekney SA (2014). Precision breeding of grapevine (Vitis vinifera L.) for improved traits. Plant science..

[5] Iocco P, Franks T, Thomas MR (2001). Genetic transformation of major wine grape cultivars of Vitis vinifera L. Transgenic research..

[6] Stamp JA, Colby SM, Meredith CP (1990). Direct shoot organogenesis and plant regeneration from leaves of grape (Vitis spp.). Plant Cell, Tissue and Organ Culture..

[7] Tao JM, Zhang ZM, Zhang Z, Geng QF, Cai BH (2005). Study on plant regeneration from *in vitro* culture of Vitis vinifera cv. Manicure Finger [J]. Journal of Fruit Science..

[8] Li JF, Zhang Z, Zhuang ZM, Tong ZG, Tao JM (2007). Plant regeneration of grape rootstock ‘5BB’ (Vitis berlandieri × V. riparia) *in vitro*. Acta Bot. Boreal. –Occident. Sin..

[9] Zhang P, Yu ZY, Cheng ZM, Zhang Z, Tao JM (2011). *In vitro* explants regeneration of the grape Wink (Vitis vinifera L. Wink). Journal of Plant Breeding and Crop Science..

[10] Nicholson KL, Tarlyn N, Armour T, Swanson ME, Dhingra A (2012). Effect of phyllotactic position and cultural treatments toward successful direct shoot organogenesis in dwarf ‘Pixie’grapevine (Vitis vinifera L.). Plant Cell, Tissue and Organ Culture..

[11] Barlass M, Skene KGM (1980). Studies on the fragmented shoot apex of grapevine: I. The regenerative capacity of leaf primordial fragments *in vitro*. Journal of Experimental Botany..

[12] Dutt M, Li ZT, Dhekney SA, Gray DJ (2007). Transgenic plants from shoot apical meristems of Vitis vinifera L.“Thompson Seedless” via *Agrobacterium*-mediated transformation. Plant cell reports..

[13] Rajasekaran K, Mullins MG (1981). Organogenesis in internode explants of grapevines. Vitis..

[14] Bayir A, Uzun HI, Yalcin EA (2005). Effect of Genotype on Callus Formation And Organogenesis in Vitis. In “International Workshop on Advances in Grapevine and Wine Research”..

[15] Mezzetti B, Pandolfini T, Navacchi O, Landi L (2002). Genetic transformation of Vitis vinifera via organogenesis. BMC biotechnology..

[16] Rotino GL, Perri E, Zottini M, Sommer H, Spena A (1997). Genetic engineering of parthenocarpic plants. Nature biotechnology..

[17] Pandolfini T, Rotino GL, Camerini S, Defez R, Spena A (2002). Optimisation of transgene action at the post-transcriptional level: high quality parthenocarpic fruits in industrial tomatoes. BMC biotechnology..

[18] Costantini E (2007). Auxin synthesis-encoding transgene enhances grape fecundity. Plant physiology..

[19] Bertsch C (2005). Genetic chimerism of Vitis vinifera cv. Chardonnay 96 is maintained through organogenesis but not somatic embryogenesis. BMC Plant Biology..

[20] Xie X, Agüero CB, Wang Y, Walker MA (2016). Genetic transformation of grape varieties and rootstocks via organogenesis. Plant Cell, Tissue and Organ Culture..

[21] Pérez-Jiménez M, Carrillo-Navarro A, Cos-Terrer J (2012). Regeneration of peach (Prunus persica L. Batsch) cultivars and Prunus persica × Prunus dulcis rootstocks via organogenesis. Plant Cell, Tissue and Organ Culture..

[22] Sabbadini, S., Pandolfini, T., Girolomini, L., Molesini, B., Navacchi, O. Peach (Prunus persica L.). (ed. Wang, K.) *Agrobacterium* Protocols. (Springer, New York, 2015), pp 205–215. 10.1007/978-1-4939-1658-0_17 25416260

[23] Cappelletti R, Sabbadini S, Mezzetti B (2016). The use of TDZ for the efficient *in vitro* regeneration and organogenesis of strawberry and blueberry cultivars. Scientia horticulturae..

[24] Rakosy-Tican E (2007). The usefulness of the gfp reporter gene for monitoring *Agrobacterium*-mediated transformation of potato dihaploid and tetraploid genotypes. Plant cell reports..

[25] Zhou Q (2014). A circulatory system useful both for long-term somatic embryogenesis and genetic transformation in Vitis vinifera L. cv. Thompson Seedless. Plant Cell, Tissue and Organ Culture..

[26] Perl A, Lotan O, Abu-Abied M, Holland D (1996). Establishment of an *Agrobacterium*-mediated transformation system for grape (Vitis vinifera L.): the role of antioxidants during grape–*Agrobacterium* interactions. Nature Biotechnology..

[27] De La Riva GA, González-Cabrera J, Vázquez-Padrón R, Ayra-Pardo C (1998). *Agrobacterium* tumefaciens: a natural tool for plant transformation. Electronic Journal of Biotechnology..

[28] Li ZT (2006). Optimizing *Agrobacterium*-mediated transformation of grapevine. Vitro Cellular & Developmental Biology-Plant..

[29] Maximova SN, Dandekar AM, Guiltinan MJ (1998). Investigation of *Agrobacterium*-mediated transformation of apple using green fluorescent protein: high transient expression and low stable transformation suggest that factors other than T-DNA transfer are rate-limiting. Plant molecular biology..

[30] Dominguez A, Fagoaga C, Navarro L, Moreno P, Peña L (2002). Regeneration of transgenic citrus plants under non selective conditions results in high-frequency recovery of plants with silenced transgenes. Molecular Genetics and Genomics..

[31] Ballester A, Cervera M, Pena L (2008). Evaluation of selection strategies alternative to nptII in genetic transformation of citrus. Plant cell reports..

[32] Yau YY, Davis SJ, Ipek A, Simon PW (2008). Early identification of stable transformation events by combined use of antibiotic selection and vital detection of green fluorescent protein (GFP) in carrot (Daucus carota L.) callus. Agricultural sciences in China..

[33] Kaeppler HF, Menon GK, Skadsen RW, Nuutila AM, Carlson AR (2000). Transgenic oat plants via visual selection of cells expressing green fluorescent protein. Plant cell reports..

[34] Cervera M, Navarro A, Navarro L, Peña L (2008). Production of transgenic adult plants from clementine mandarin by enhancing cell competence for transformation and regeneration. Tree physiology..

[35] Rosellini D (2012). Selectable markers and reporter genes: a well furnished toolbox for plant science and genetic engineering. Critical reviews in plant sciences..

[36] Wei Z, Wang X, Xing S (2012). Current progress of biosafe selectable markers in plant transformation. Journal of Plant Breeding and Crop Science..

[37] Breyer D, Kopertekh L, Reheul D (2014). Alternatives to antibiotic resistance marker genes for *in vitro* selection of genetically modified plants–scientific developments, current use, operational access and biosafety considerations. Critical reviews in plant sciences..

[38] Das D, Reddy M, Upadhyaya K, Sopory S (2002). An efficient leaf-disc culture method for the regeneration via somatic embryogenesis and transformation of grape (Vitis vinifera L.). Plant Cell Reports..

[39] Li ZT, Dhekney SA, Dutt M, Gray DJ (2008). An improved protocol for *Agrobacterium*-mediated transformation of grapevine (Vitis vinifera L.). Plant Cell, Tissue and Organ Culture..

[40] Li ZT, Kim KH, Jasinski JR, Creech MR, Gray DJ (2012). Large-scale characterization of promoters from grapevine (Vitis spp.) using quantitative anthocyanin and GUS assay systems. Plant science..

[41] Kandel R (2016). Evaluation of a grapevine-derived reporter gene system for precision breeding of Vitis. Plant Cell, Tissue and Organ Culture..

[42] Scorza R (1996). Producing TransgenicThompson Seedless’ Grape (Vitis vinifera L.) Plants. Journal of the American Society for Horticultural Science..

[43] Wang Q (2005). Improvement of *Agrobacterium*-mediated transformation efficiency and transgenic plant regeneration of Vitis vinifera L. by optimizing selection regimes and utilizing cryopreserved cell suspensions. Plant Science..

[44] Dhekney SA, Li ZT, Gray DJ (2011). Grapevines engineered to express cisgenic Vitis vinifera thaumatin-like protein exhibit fungal disease resistance. In vitro Cellular & Developmental Biology-Plant..

[45] Dabauza M (2015). Enhanced resistance to Botrytis cinerea in genetically-modified Vitis vinifera L. plants over-expressing the grapevine stilbene synthase gene. Plant Cell, Tissue and Organ Culture..

[46] Li ZT, Hopkins DL, Gray DJ (2015). Overexpression of antimicrobial lytic peptides protects grapevine from Pierce’s disease under greenhouse but not field conditions. Transgenic research..

[47] Clog E, Bass P, Walter B (1990). Plant regeneration by organogenesis in Vitis rootstock species. Plant cell reports..

[48] Torregrosa L, Bouquet A (1996). Adventitious bud formation and shoot development from *in vitro* leaves of Vitis x Muscadinia hybrids. Plant cell, tissue and organ culture..

[49] Martinelli L, Poletti V, Bragagna P, Poznanski E (1996). A study on organogenic potential in the Vitis genus. Vitis..

[50] Amar AB (2007). Efficient procedure for grapevine embryogenic suspension establishment and plant regeneration: role of conditioned medium for cell proliferation. Plant cell reports..

[51] Gambino G, Ruffa P, Vallania R, Gribaudo I (2007). Somatic embryogenesis from whole flowers, anthers and ovaries of grapevine (Vitis spp.). Plant Cell, Tissue and Organ Culture..

[52] Torregrosa L, Lopez G, Bouquet A (2017). Antibiotic Sensitivity of Grapevine: A Comparison Between the Effect of Hygromycin and Kanamycin on Shoot Development of Transgenic 110 Richter Rootstock (Vi tis B erlandieri x Vi tis rupestris). South African Journal of Enology and Viticulture..

[53] Colby SM, Juncosa AM, Meredith CP (1991). Cellular differences in *Agrobacterium* susceptibility and regenerative capacity restrict the development of transgenic grapevines. Journal of the American Society for Horticultural Science..

[54] Aguero CB, Meredith CP, Dandekar AM (2006). Genetic transformation of Vitis vinifera L. cvs Thompson Seedless and Chardonnay with the pear PGIP and GFP encoding genes. Vitis-Geilweilerhof.

[55] Kurmi US (2011). Plant regeneration of Vitis vinifera (L) via direct and indirect organogenesis from cultured nodal segments. J Agric Technol..

[56] Pena L, Perez RM, Cervera M, Juarez JA, Navarro L (2004). Early events in *Agrobacterium*‐mediated genetic transformation of citrus explants. Annals of Botany..

[57] Del Pozo JC, Lopez-Matas MA, Ramirez-Parra E, Gutierrez C (2005). Hormonal control of the plant cell cycle. Physiologia Plantarum..

[58] Arias RS, Filichkin SA, Strauss SH (2006). Divide and conquer: development and cell cycle genes in plant transformation. TRENDS in Biotechnology..

[59] López-Pérez AJ, Velasco L, Pazos-Navarro M, Dabauza M (2008). Development of highly efficient genetic transformation protocols for table grape Sugraone and Crimson Seedless at low *Agrobacterium* density. Plant Cell, Tissue and Organ Culture..

[60] Ozden M, Karaaslan M (2011). Effects of cytokinin on callus proliferation associated with physiological and biochemical changes in Vitis vinifera L. Acta Physiologiae Plantarum..

[61] Murashige T, Skoog F (1962). A revised medium for rapid growth and bio assays with tobacco tissue cultures. Physiologia plantarum..

