# Supplementary material for: Comparison of regeneration capacity and Agrobacterium-mediated cell transformation efficiency of different cultivars and rootstocks of Vitis spp. via organogenesis
Source: Sci Rep. 2019 Jan 24;9:582. doi: 10.1038/s41598-018-37335-7 (PMC6345860; doi:10.1038/s41598-018-37335-7)
Supplement: Supplementary file 1 — Supplementary Figures [file 41598_2018_37335_MOESM1_ESM.pdf]

Comparison of regeneration capacity and *Agrobacterium*-mediated cell transformation efficiency of different cultivars and rootstocks of *Vitis* spp. via organogenesis

Sabbadini S.[1], Capriotti L.[1], Molesini B.[2], Pandolfini T.[2], Navacchi O.[3], Limera C.[1], Ricci A.[1], Mezzetti B.[1*]

[1]Department of Agricultural, Food and Environmental Sciences, Università Politecnica delle Marche, Ancona, Italy.
[2]Department of Biotechnology, University of Verona, Italy.
[3]Vitroplant International, Cesena, Italy.

*Email: b.mezzetti@staff.univpm.it

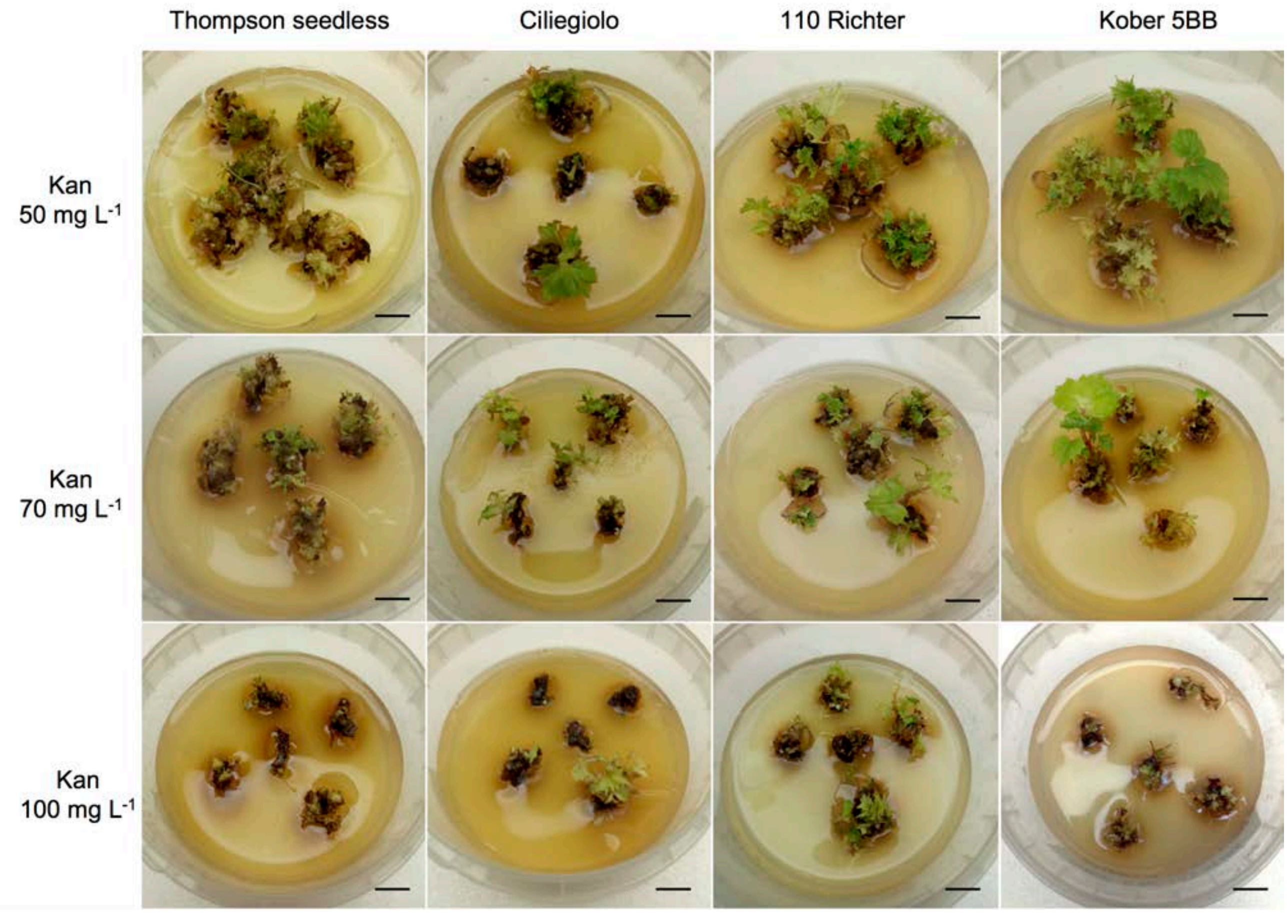

Supplementary Fig.1 Sensitivity test to kanamycin of non-transformed MB slices of Thompson seedless, Ciliegiolo, 110 Richter and Kober 5BB. A representative combined image after three weeks of culture on IM3 supplemented with increasing kanamycin concentrations (50 mg L^{-1}, 70 mg L^{-1}, and 100 mg L^{-1}).

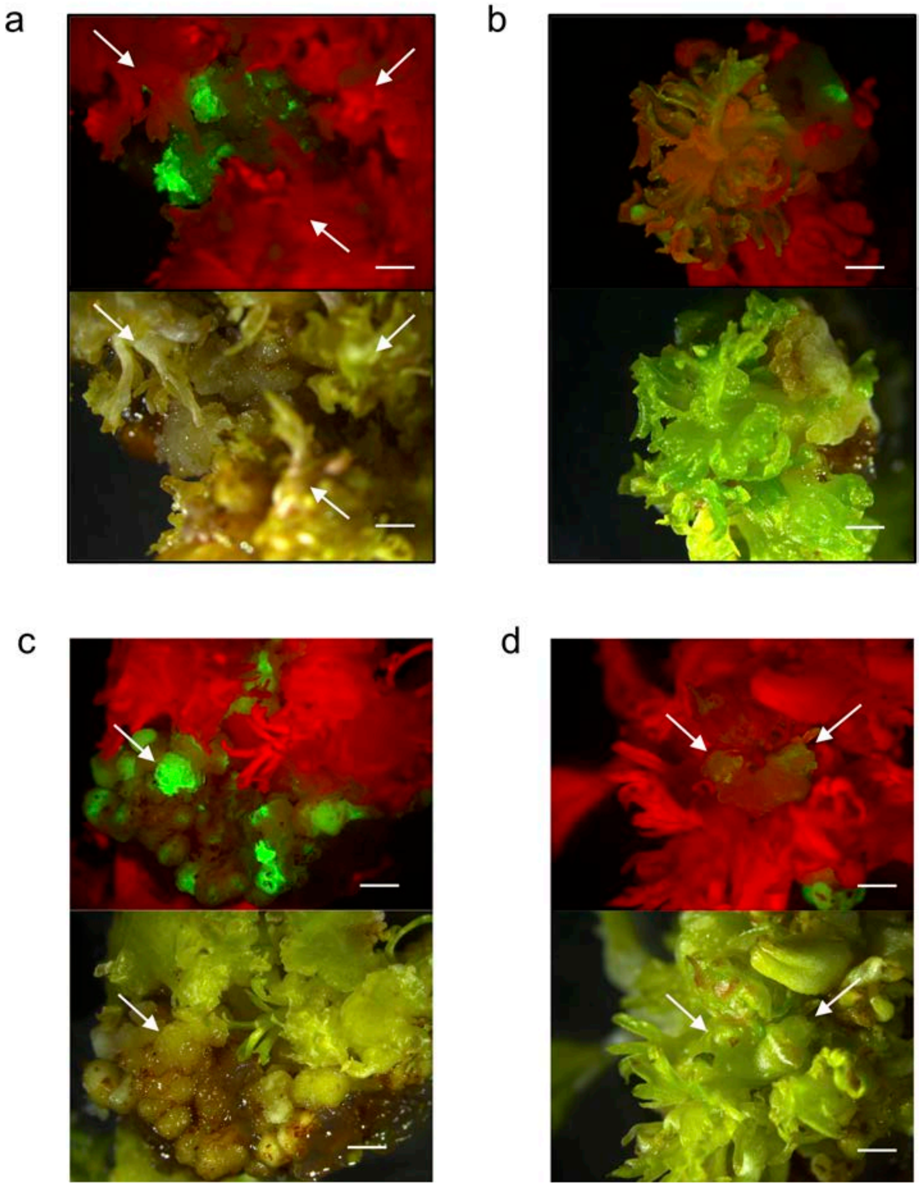

Supplementary Fig.2 Thompson seedless MB slices. Uniform fluorescence with bright green colour was observed in transformed tissues under UV light: a) regeneration of escapes (non-transformed shoots) close to transgenic cells after 9 weeks of culture at kanamycin 70 mg L^{-1}, white arrows indicate groups of regenerating escapes; b) eGFP expressing shoot regenerated after 9 weeks of culture on kanamycin-free medium; c) White arrows indicate a eGFP fluorescent globular callus with no regenerative competence after 3 weeks of culture with kanamycin 70 mg L^{-1}; d) eGFP-fluorescent adventitious shoots regenerating after 3 weeks of culture with kanamycin 70 mg L^{-1}, white arrows indicate a translucent meristematic cells cluster. Upper and lower panels show images taken under UV and white light, respectively (*bar*= 2mm).

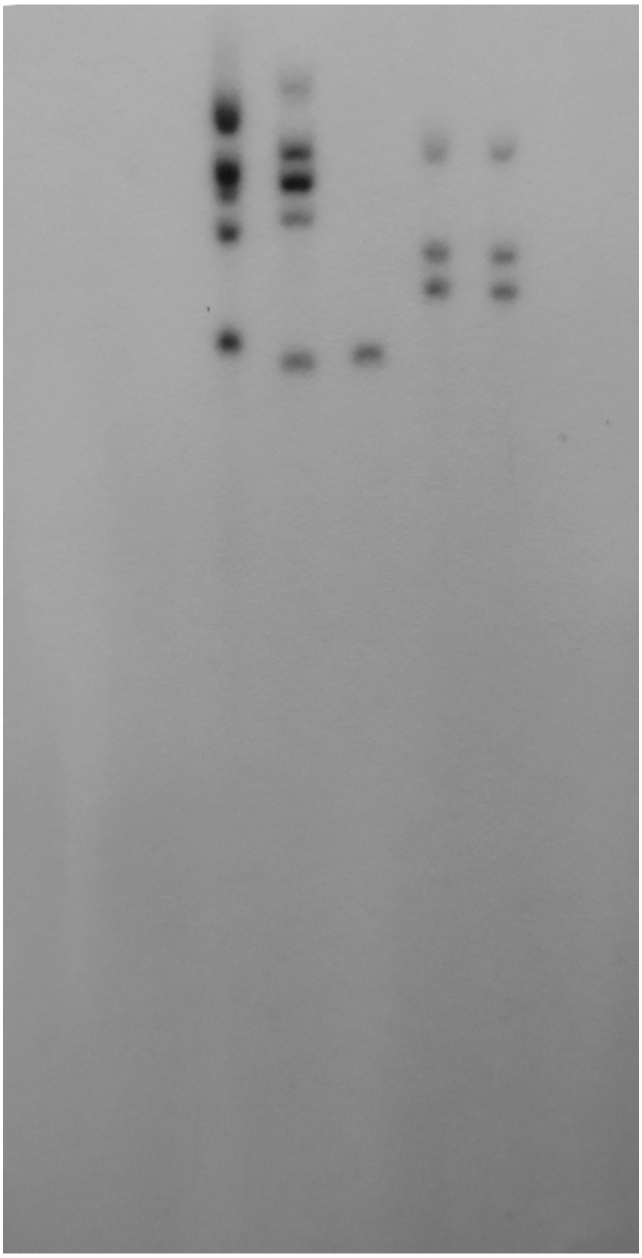

Supplementary Fig.3 Full-length Southern blot shown in Fig.4 d.
